# Need estimates of psychiatric beds: a systematic review and meta-analysis

**DOI:** 10.1017/S0033291724002307

**Published:** 2024-10

**Authors:** Adrian P. Mundt, Enzo Rozas-Serri, Francesco D. Fritz, Sabine Delhey, Mathias Siebenförcher, Stefan Priebe

**Affiliations:** 1 Medical Faculty, Universidad Diego Portales, Santiago, Chile; 2 Departamento de Neurología y Psiquiatría, Clínica Alemana de Santiago, Facultad de Medicina Clínica Alemana, Universidad del Desarrollo, Santiago, Chile; 3 Department of Mental Health, Hospital Clínico Universidad de Chile, Santiago, Chile; 4 Departamento de Neurología y Psiquiatría, Clínica Alemana de Santiago, Facultad de Medicina Clínica Alemana, Universidad del Desarrollo, Santiago, Chile; 5 Department of Psychiatry and Psychotherapy Campus Mitte, Charité Universitätsmedizin Berlin, Germany; 6 Medical Faculty, Universidad Diego Portales, Santiago, Chile; 7 Department of Psychiatry and Psychotherapy Campus Mitte, Charité Universitätsmedizin, Berlin, Germany; 8 Centre for Mental Health Research, City, University of London, London, UK

**Keywords:** deinstitutionalization, meta-analysis, need estimates, psychiatric beds, systematic review

## Abstract

This study aimed to review and synthesize the need estimates for psychiatric beds, explore how they changed over time and compare them against the prevalence of actually existing beds. We searched PubMed, Embase classic and Embase, PsycINFO and PsycIndex, Open Grey, Google Scholar, Global Health EBSCO and Proquest Dissertations, from inception to September 13, 2022. Publications providing estimates for the required number of psychiatric inpatient beds were included. Need estimates, length of stay, and year of the estimate were extracted. Need estimates were synthesized using medians and interquartile ranges (IQRs). We also computed prevalence ratios of the need estimates and the existing bed capacities at the same time and place. Sixty-five publications with 98 estimates were identified. Estimates for bed needs were trending lower until 2000, after which they stabilized. The twenty-six most recent estimates after 2000 were submitted to data synthesis (*n* = 15 for beds with unspecified length of stay, *n* = 7 for short-stay, and *n* = 4 for long-stay beds). Median estimates per 100 000 population were 47 (IQR: 39 to 50) beds with unspecified length of stay, 28 (IQR: 23 to 31) beds for short-stay, and 10 (IQR: 8 to 11) for long-stay beds. The median prevalence ratio of need estimates and the actual bed prevalence was 1.8 (IQR: 1.3 to 2.3) from 2000 onwards. Historically, the need estimates for psychiatric beds have decreased until about 2000. In the past two decades, they were stable over time and consistently higher than the actual bed numbers provided.

## Introduction

Psychiatric reforms in the second half of the past century were guided by human rights concerns in light of often unacceptable living conditions in asylums at the time, and a call for more social inclusion of people with mental illness (Chow & Priebe, 2013; Lamb & Bachrach, 2001; Novella, 2010). This process, referred to as ‘deinstitutionalization’ (Thornicroft & Bebbington, 1990), led to a reduction of psychiatric bed numbers and the development of community-based services. Currently, psychiatric bed prevalence varies greatly between countries and geographical areas (Blüml, Waldhor, Kapusta, & Vyssoki, 2015; Mundt et al., 2021b; Siebenförcher et al., 2022). Countries with lower per capita income usually have fewer psychiatric beds (Mundt et al., 2022a). While the median number of psychiatric beds in high-income countries (HICs) is around 44 per 100 000 population, it is only seven in low-and-middle-income countries (LMICs). Residential care facilities are also much rarer in LMICs as compared to HICs. In HICs, there are on average 25 residential care beds per 100 000 population overall, and specifically in Europe there are 53 (World Health Organization, 2021). Deinstitutionalization policies did not establish a minimum or optimum number of required beds to sustain a balanced and effective mental health care system.

Whether the number of psychiatric beds should be reduced even further or be kept at the current levels, or whether the bed reduction has gone too far and more beds should be provided, is the subject of ongoing debate (Guaiana, Bastiampillai, Allison, & O'Reilly, 2019; Mundt et al., 2015; Ose, Kalseth, Ådnanes, Tveit, & Lilleeng, 2018; Tyrer, 2011). Effective psychiatric bed planning should be informed by history and based on prevalence data, need estimates, and comparisons across countries and regions.

The term ‘psychiatric bed’ typically includes different types of mental health inpatient facilities or treatment places. A definition based on the Mental Health Atlas Project (World Health Organization, 2021) includes mental hospital short- and long-stay inpatient services, beds in general hospital psychiatric units (GHPU), community-based psychiatric inpatient places and forensic inpatient units. The definition also includes both public and private facilities, specialized beds for children and adolescents and those for other specific target groups (e.g. old age). It excludes facilities that exclusively treat people with substance use disorders or intellectual disability, community residential facilities and services exclusively providing recovery and rehabilitation. However, the definitions used in the literature are not consistent across countries and sometimes do not consider long-stay units and private psychiatric hospitals (Guaiana et al., 2019; OECD Health Statistics, 2023).

Three approaches have been used to estimate the appropriate number of psychiatric beds (O'Reilly, Allison, & Bastiampiallai, 2019). Firstly, an empirical population health approach estimates how many beds are needed for a given catchment area based on epidemiological, service and health outcome data (Harris, Buckingham, Pirkis, Groves, & Whiteford, 2012). Secondly, a normative approach assumes that different catchment areas with similar demographic and mental health policies will require a similar number of psychiatric beds (Harris et al., 2012; O'Reilly et al., 2019). Thirdly, estimates can be based on expert opinion and consensus approaches (Fuller Torrey, Entsminger, Geller, Stanley, & Jaffe, 2008; Müller, 1973). Recently, a stepwise combination of several approaches has been recommended for policy planning in the United States (US) (McBain, Cantor, & Eberhart, 2022a). Considering all three approaches in this systematic review, we aimed to review and synthesize the published estimates for appropriate psychiatric bed numbers, explore how they changed over time and compare them against actual bed capacities.

## Methods

### Search strategy and selection criteria

For this systematic review and meta-analysis, we searched seven databases from their inception until September 13, 2022: PubMed, Embase classic and Embase, PsycINFO and PsycIndex, Open grey, Google Scholar, Global Health EBSCO and Proquest Dissertations. We used the string ‘psychiatric AND hospital* AND bed*[Title/Abstract]’ with no filters based on study types or language. We restricted searches in PubMed, PsycINFO and PsycIndex to title and abstract. The historical perspective was included to identify and describe time trends of the need estimates. The timing of psychiatric reforms aiming to strengthen outpatient care and reduce bed capacities varied substantially across countries and regions. Since Google Scholar produces very high numbers of hits (>500 000) and sorts them by relevance, the search in this database was initially limited to 500 hits and with the updated search to 666 hits. This was to ensure that the psychiatric beds were a central topic of the articles rather than tangentially discussed. We reviewed references and citations of articles retained in this study for additional unidentified publications.

Scientific publications providing estimates of the required number of psychiatric inpatient beds were included. Studies not providing numerical estimates and those referring only to beds or places in other mental health facilities such as day hospitals or residential facilities were excluded. Publications providing numerical estimates of psychiatric beds exclusively for specific populations (e.g. schizophrenia, only old age, only children and adolescents) were also excluded since those estimates refer only to a proportion of all psychiatric beds. Duplicate reports referring to the same estimate were excluded.

Data were independently extracted by MS, SD, and ERS. Any differences between reviewers were discussed within the research team to find a consensus. The primary outcome extracted was the estimate of the need for psychiatric beds per 100 000 population. If the need estimate was given as an unstandardized number, the population size of the catchment area for the same year was extracted from the World Bank for whole countries or other online sources for more local or regional catchment areas. Estimates were grouped into estimates for (1) the total number of psychiatric beds without specification of the length of stay, which from here onwards are referred to as unspecified length of stay; (2) beds for acute inpatient, short- and up to medium-stay, and general hospital psychiatric units (GHPU); (3) long-stay psychiatric beds that may or may not include medium lengths of stay, including the traditional long-stay units in psychiatric hospitals and new long-stay facilities according to the terminology used by the authors.

The following variables were extracted for all included studies: year of the estimate and year of the publication; whether the estimate referred to a local, national or international geographical area (reference or catchment area); the country of the estimate; the income group of the country based on World Bank categories (The World Bank, 2023); and the approach used to support the estimate (population health approach, normative approach, expert consensus or opinion). Population sizes were retrieved for the same year as the estimates. Actual bed numbers for the corresponding catchment area were also extracted from the studies, where available.

### Data analyses

All estimates were calculated as numbers per 100 000 population to facilitate comparisons and analysis. When no such prevalence was provided, the numbers per 100 000 population were calculated based on the number of beds estimated and the population size of the given catchment area or country and year. For the estimates reported as ranges, the central point of the range was used for data synthesis. Decimals were rounded to the nearest whole number. Confidence intervals (CI) were calculated for the prevalence, taking into account the population size for which the estimate was made in the same year. We pooled bed need estimates separately for the three lengths of stay (unspecified, short-stay, and long-stay) using random-effects meta-analyses and calculated 95% confidence intervals (CI) for the pooled values. The I^2^-statistic was used to report the heterogeneity between studies. Analyses were conducted with STATA by Stata Corp. For the trend analyses, the year of the estimate was considered. In forecasting studies, the year of publication was used. Trendlines were visualized using Excel, Microsoft Corp. Given the low number of estimates and the very high heterogeneity between studies, the more recent estimates from 2000 onwards were assessed by calculating the median and interquartile ranges. We also stratified the need estimates for beds with unspecified length of stay after 2000 by the method that was used for the estimate (epidemiological, expert consensus/opinion, and normative approach).

The actual bed prevalence was extracted from the papers and, if not available from the publication, we used data from the World Health Organization Atlas Project. The actual psychiatric bed prevalence for the closest available year was considered, if not available for the same year of the estimate. We calculated prevalence ratios of the need estimates and the actual prevalence of beds available in the given reference area or country. This reflects to what extent the need estimates differed from the actual number of psychiatric beds at that time. We calculated 95% CI of the prevalence ratios taking into account the population size. Meta-analyses and meta-regression with the year of the estimate as independent variables were conducted with STATA. Trends of prevalence ratios for the different categories of beds were visualized with Excel, Microsoft Corp. The more recent bed prevalence and prevalence ratios from 2000 onwards were assessed by calculating descriptive statistics, the median and interquartile ranges.

We scored the quality of the estimates based on whether the publication was in a peer-reviewed journal and whether the estimates were based on epidemiological data. We also established whether the publication had the primary objective of reporting a need estimate for psychiatric beds or not; whether the estimate was based on a gross number of beds or a prevalence; whether it was a single specific number or a range; whether it referred to a specific or unspecified length of stay; and whether authors explicitly specified which minimum or optimal number of beds would be appropriate.

## Results

**Figure 1.**
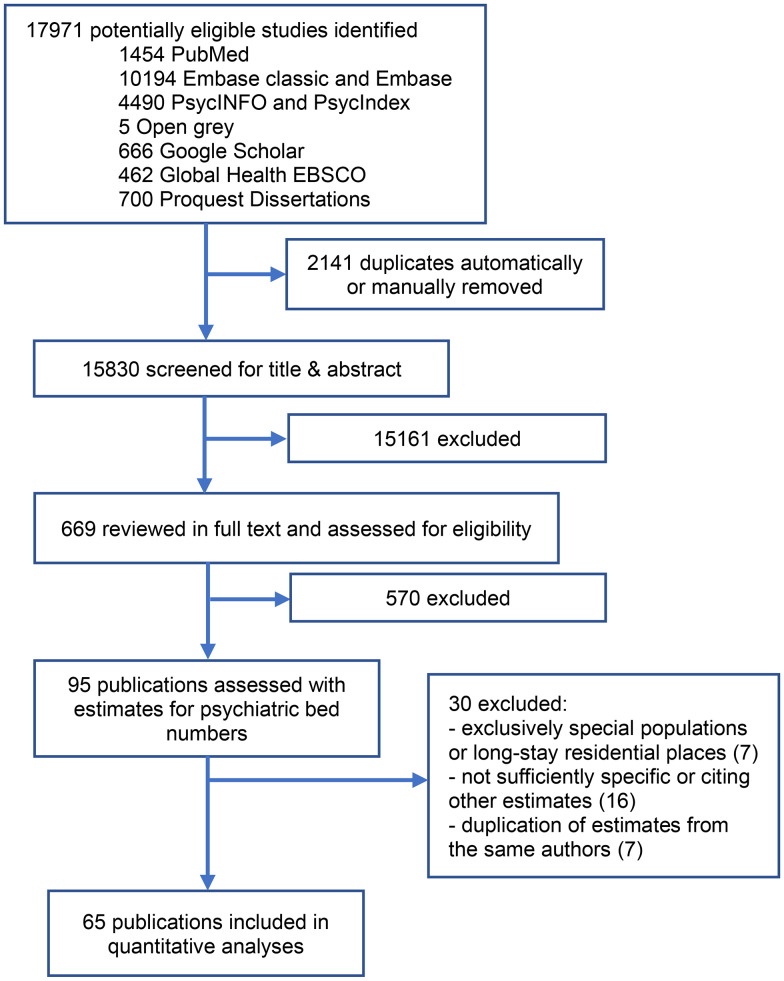
Study selection flowchart following PRISMA.

We identified 17 971 papers. After removal of duplicates and title/abstract screening, 669 were retained for full-text revision. Among those, 95 publications contained estimates for the appropriate number of psychiatric beds. Thirty were excluded because they were duplicate publications of the same authors without providing new data or methodologies, or because they cited an estimate rather than making an original one, or because they referred only to special populations or long-stay residential facilities (Fig. 1). Sixty-five studies met the inclusion criteria, providing 98 estimates. For Ramsay et al. (1997), Strathdee & Thornicroft (1992) and Wing (1992), data were retrieved from the same textbook (Wing, Brewin, & Thornicroft, 2001). Also, Bachrach (Bachrach, 1975) compiled six publications that we could not directly access. The estimate provided by Robertson (1981) was extracted from Hirsch (1983).

Fourteen publications (Bachrach, 1975; Boardman & Parsonage, 2007; Dillard & Fillion, 1998; Fryers, 1974; Gore, Jones, Taylor, & Ward, 1964; Häfner, 1987; Harris et al., 2012; Lund & Flisher, 2006; Lund, Flisher, Porteus, & Lee, 2002; Mezey & Syed, 1972; New Zealand Mental Health Commission, 1998; Oldham, 1969; Royal Commission into Victoria's Mental Health System, 2019; Tooth & Brooke, 1961; Trant et al., 1984) provided estimates for all three categories of length of stay (unspecified, short and long-stay) and four (Richman & Kennedy, 1965; Robertson, 1981; Straathof, 1976; UK Department of Health & Social Security, 1975) reported numbers for two of the three types. Forty-eight papers reported numbers for only one of the three categories. Thus, 98 estimates were included: 39 for psychiatric beds with unspecified length of stay, 34 for short-stay beds, and 25 for long-stay beds (Table 1).

**Table 1.** Publications of need estimates for psychiatric beds per 100 000 population with year, country, income group, length of stay and approach of the estimate

Author (year of publication)	Year of the estimate	Country	Income group	Reference area of the estimate [catchment area or jurisdiction when local]	Estimates for psychiatric beds per 100 000 population			Approach of the estimate					
								Epidemiological data		Expert		Normative	
					Unspecified length of stay (N 39)	Short-stay (N 34)	Long-stay (N 24)	Current epidemiological data	Epidemiological data forecast	Expert consensus	Expert opinion	Normative non-scientific reports	Normative government guidelines
Strömgren (1958)	1958	Denmark	HI	National	250	ND	ND	X					
Tooth and Brooke (1961)	1959	UK	HI	National [England & Wales]	180	89	91	X					
Gore et al. (1964)	1975	UK	HI	Local [Leeds region]	193	56	137		X				
Richman and Kennedy (1965)	1971	Canada	HI	National	197	47	ND		X				
Ohio Department of Mental Hygiene and Correction (1965)^1^	1965	USA	HI	Local [Ohio]	280	20	260					X	
Maryland Board of Health and Mental Hygiene (1965)^1^	1965	USA	HI	Local [Maryland]	375	ND	ND					X	
Valdés (1966)	1966	Spain	HI	National	200	ND	ND				X		
Orwin (1967)	1972	UK	HI	Local [Birmingham]	ND	ND	36		X				
California Department of Mental Hygiene (1968)^1^	1976	USA	HI	Local [California]	65	ND	ND					X	
Baker (1969)	1969	UK	HI	Local [St Mary Abbot's Hospital, London]	ND	50	ND	X					
Oldham (1969)	1987	UK	HI	Local [London Borough of Southwark]	134	69	65		X				
Hoenig and Hamilton (1969)	1969	UK	HI	Local [Manchester]	ND	40	ND					X	
Crane & Gorwic (1970)^1^	2000	USA	HI	Local [Montgomery County, Maryland]	ND	100	ND		X				
*Cross, Hassall, and Spencer* (1970)	1970	UK	HI	Local [Worcestershire]	ND	ND	65	X					
Hailey (1971)	1971	UK	HI	Local [Camberwell, London]	ND	ND	88	X					
*UK Department of Health and Social Security* (*Wing*, 1971)	1969	UK	HI	National	50	ND	ND						X
Mezey and Syed (1972)	1985	UK	HI	Local [north-east London]	99	47	52		X				
Lipscomb (1972)	1972	Canada	HI	Local [Yorkton]	60	ND	ND	X					
Lawrence (1972)^1^	1972	USA	HI	Local [District of Columbia]	ND	50	ND				X		
Lawson (1972)	1972	Australia	HI	National	180	ND	ND				X		
Brown (1973)^1^	1972	USA	HI	Local [Multiple sites]	ND	ND	33^†^	X					
Müller (1973)	1973	Switzerland	HI	International [developed countries]	100	ND	ND			X			
Fryers (1974)	1982	UK	HI	Local [Salford]	230	64	166		X				
*UK Department of Health and Social Security (DHSS)* (1975)	1975	UK	HI	National	ND	50	17						X
Straathof (1976)	1976	Netherlands	HI	National	177	27	ND	X					
Robertson (1981)^2^	1991	UK	HI	National	140	ND	53		X				
Hirsch (1983)	1983	UK	HI	National [England & Wales]	ND	54	ND	X					
*McCreadie, Wilson, and Burton* (1983)	1983	UK	HI	National [Scotland]	ND	ND	20	X					
Goplerud (1986)	1981	USA	HI	National [Metropolitan areas]	ND	48	ND	X					
*Richman, Boutilier, and Harris* (1984)	1984	Canada	HI	National [Atlantic provinces of Canada]	ND	27	ND	X					
Gudeman and Shore (1984)	1984	USA	HI	Local [Massachusetts]	ND	ND	15	X					
Trant et al. (1984)	1984	Ireland	HI	National	100	50	50						X
Häfner (1987)	1987	Germany	HI	International [developed countries]	110	65	45	X					
Hirsch (1987)	1987	UK	HI	National [England & Wales]	ND	44	ND	X					
Andrews (1990)	1989	Australia	HI	National	35	ND	ND	X					
*Holloway, Silverman, and Wainwright* (1992)	1992	UK	HI	Local [East Lambeth, London]	ND	99	ND	X					
Strathdee & Thornicroft (1992)^3^	1992	UK	HI	National	ND	40	ND	X					
Wing (1992)^3^	1992	UK	HI	National	ND	40	ND	X					
Pridmore and Jones (1992)	1992	Australia	HI	Local [Tasmania]	ND	ND	5	X					
Rosenman (1995)	1995	Australia	HI	Local [NSW & ACT]	ND	16	ND	X					
*World Health Organization* (1996)	1996	International	LMI	International [developing countries]	10	ND	ND					X	
Trieman and Leff (1996)	1996	UK	HI	Local [London]	ND	ND	11	X					
Gordon (1997)	1997	Canada	HI	National	50	ND	ND					X	
Ramsay et al. (1997)^3^	1997	UK	HI	Local [London]	ND	45	ND	X					
Dillard and Fillion (1998)	1998	Canada	HI	Local [Quebec]	40	25	15						X
Disley et al., on behalf of the New Zealand Mental Health Commission (1998)	1998	New Zealand	HI	National	41	15	12						X
Audini, Duffett, Lelliott, Pearce, and Ayres (1999)	1999	UK	HI	Local [Inner London]	ND	60	ND	X					
Gebhardt and Schmidt-Michel (2002)	2002	Germany	HI	Local [Ravensburg]	ND	33	ND	X					
Lesage et al. (2002)	2002	Canada	HI	Local [East-end Montreal]	ND	18	ND	X					
Lund and Flisher (2006)	2006	South Africa	UMI	National	38	28	10	X					
Boardman and Parsonage (2007)	2011	UK	HI	National [England & Wales]	48	30	0		X				
*Leite Gastal* et al. (2007)	2007	Brazil	UMI	National	50	ND	ND						X
*Fuller Torrey* et al. (2008)	2008	USA	HI	National	50	ND	ND			X			
Myklebust et al. (2009)	2008	Norway	HI	Local [Vesterålen & Lofoten]	100	ND	ND	X					
Candiago et al. (2011)	2010	Brazil	UMI	Local [Rio Grande do Sul]	22	ND	ND	X					
Tyrer (2011)	2011	UK	HI	National	47	ND	ND				X		
Harris et al. (2012)	2012	Australia	HI	Local [Queensland]	30	20	10	X					
La et al. (2016)	2015	USA	HI	Local [North Carolina]	39	ND	ND	X					
Allison et al. (2018)	2018	Australia	HI	International	50	ND	ND				X		
Benjamin et al. (2018)	2018	Australia	HI	Local [Hobart, South Tasmania]	ND	26	ND				X		
*Royal Commission into Victoria's Mental Health System* (2019)	2019	Australia	HI	Local [Victoria]	51	30	15						X
Hudson (2020)	2015	USA	LMI-HI	International [mean for multiple countries]	46	ND	ND	X					
Okayama et al. (2020)^††^	2040	Japan	HI	National	ND	ND	93		X				
Hudson (2021)	2021	USA	HI	National	35	ND	ND	X					
*McBain, Cantor, Eberhart, Huilgol, and Estrada-Darley* (2022b)	2021	USA	HI	Local [California]	51	ND	ND					X	
Mundt et al. (2022b)	2022	International	LMI-HI	International	45	ND	ND			X			

UK, United Kingdom; USA, United States of America; HI, High-Income; UMI, Upper Middle-Income; LMI, Low- and Lower Middle-Income.

^†^Based on mean suggestions for 4 different geographical areas. ^††^ Excluded from data synthesis due to the long-term forecast method.

1 Studies compiled in Bachrach L.L. Psychiatric Bed Needs: An. Analytical Review. National Institute of Mental Health; Rockville, MD, USA: 1975. (Bachrach, 1975).

2 Cited in Hirsch (1983).

3 Studies compiled in Thornicroft G, ed. Measuring Mental Health Needs. 2nd ed. Cambridge University Press; 2001:1-21. (Wing et al., 2001).

Sixty-two out of 65 publications referred to HIC, mostly from the United Kingdom (UK), US, Canada, and Australia (online Supplementary Table 1). Only three publications were from LMICs, i.e. from Brazil (Candiago, da Silva Saraiva, Gonçalves, & Belmonte-de-Abreu, 2011; Leite Gastal et al., 2007) and South Africa (Lund & Flisher, 2006). Three international publications also included estimates for LMICs (Hudson, 2020; Mundt et al., 2022b; World Health Organization, 1996). A report from the WHO in 1996 (World Health Organization, 1996) made estimates specifically for developing countries and provided the lowest reference for beds with unspecified length of stay (10 per 100 000 population). A more recent worldwide Delphi expert consensus process estimated a minimum of 30 psychiatric beds was required per 100 000 population and that an optimal number of beds would be 60 per 100 000 (Mundt et al., 2022b).

The study characteristics are presented in Table 1 and sorted by the year of the estimate. Twenty-seven estimates referred to countries, 33 were for local areas on a sub-national level, and six had international scope. As for the approach that supported the estimates, 43 studies were based on either current or forecasting demographic and epidemiological data (Table 1). On the other hand, 14 estimates were governmental or institutional guidelines, reflecting a normative approach. Nine estimates followed either expert consensus or expert opinion approaches. One study was excluded from further analyses because it contained an unusually long forecast (for the year 2040) (Okayama, Usuda, Okazaki, & Yamanouchi, 2020). Thus, 64 studies with 97 estimates were retained for data synthesis.

**Figure 2.**
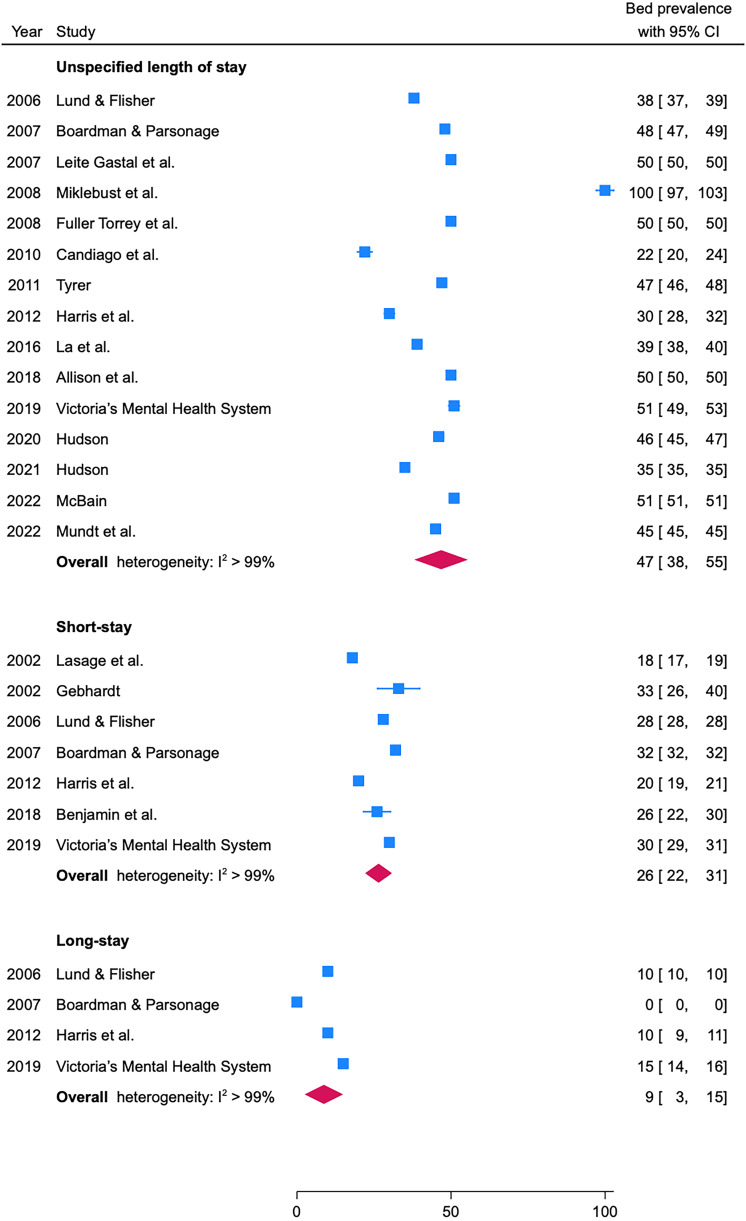
Estimates for psychiatric bed needs per 100 000 population since 2000 by the length of stay: unspecified length of stay, short-stay, and long-stay.

**Figure 3.**
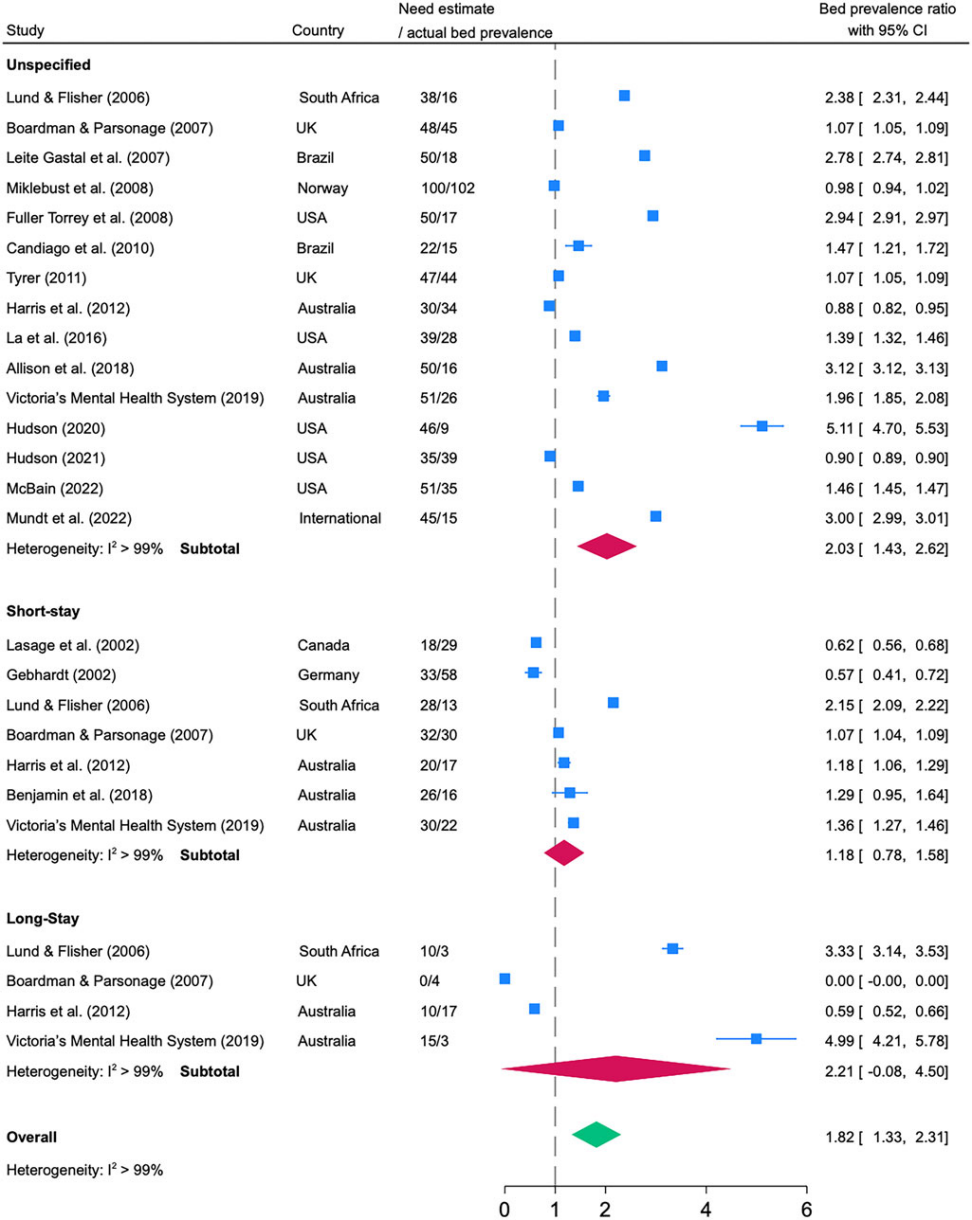
Prevalence ratios between need estimates of psychiatric beds and actual psychiatric bed provision with unspecified length of stay, short-stay, and long-stay.

For psychiatric beds with unspecified length of stay, the pooled need estimate over the entire time period was 102 (95% CI 76 to 129) per 100 000 population. Regarding short-stay beds, we found a pooled estimate of 52 (95% CI 29 to 77). For long-stay beds, the pooled estimate was 45 (95% CI 37 to 52) per 100 000 population (Online supplement, Figs 1-3). Historically, estimates of psychiatric bed needs went down over time (online Supplementary Figure 4).

We synthesized data of 26 need estimates from 2000 onwards, when those were stable over time (online Supplementary Figure 5). There were 15 need estimates for unspecified length of stay, seven for short-stay, and four for long-stay beds published after 2000. Using meta-analysis, the pooled prevalence was 47 (95% CI 38 to 55) for unspecified length of stay, 26 (95% CI 22 to 31) for short-stay, and 9 (95% CI 1.26 to 2.35) for long-stay psychiatric beds. The median need estimates were 47 (IQR: 39 to 50) beds with unspecified length of stay, 28 (IQR: 23 to 31) beds for short-stay, and 10 (IQR: 8 to 11) for long-stay beds per 100 000 population. We then stratified the 15 need estimates for beds with unspecified length of stay after 2000 by the method used for the estimate. Estimates using epidemiological data were most common (*n* = 9), with the widest range (22–100), compared to expert consensus/opinion (*n* = 3, range: 45–50), and normative approaches (*n* = 2, range: 50–51).

To assess how the need estimates related to the actual bed numbers at the same time and place, we calculated the prevalence ratios from 2000 onwards. The median time gap between need estimates and actual bed prevalence was 2.4 and a maximum of 7 years. Need estimates were generally higher than actual bed numbers (prevalence ratio >1). In the meta-analysis, the pooled value of all prevalence ratios was 1.82 (95% CI 1.33 to 2.31). With respect to the different types of psychiatric beds, the prevalence ratios were 2.03 (95% CI 1.43 to 2.62) for beds with unspecified length of stay, 1.18 (95% CI 0.78 to 1.58) for short-stay, and 2.21 (95% CI 0.00 to 4.50) for long-stay beds.

**Figure 4.**
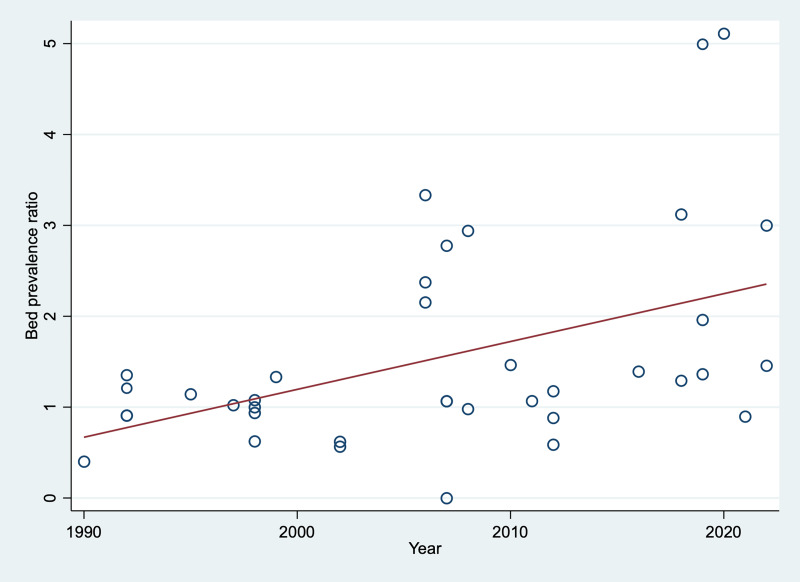
Trendline of the prevalence ratios between need estimates of psychiatric beds and actual availability over time (from 1990 onwards). Prevalence ratios smaller than 1 indicate recommendations to remove, and greater than 1, indicate recommendations to increase psychiatric bed numbers.

Historically, prevalence ratios also reflected higher estimates than actual beds. The forest plot is shown in the online Supplement Figure 6. Meta-regression analyses of all historical prevalence ratios showed a significant increase over time (*β* = 0.53, 95% CI 0.18 to 0.88; *p* = 0.004, R^2^ = 0.19). Need estimates for all types of psychiatric beds changed from being lower than the actual bed numbers (ratios of <1) to being substantially higher (ratios of >1) (Fig. 4).

Linear trendlines of the prevalence ratios for each type of psychiatric bed by length of stay are shown in online Supplement Figure 7.

## Discussion

Based on this systematic review and meta-analysis, 47 psychiatric beds with unspecified length of stay are estimated after the year 2000 to be needed per 100 000 population. Prevalence ratios show that the number of required psychiatric beds since 2000 has been estimated to be about 80% higher than the actual bed numbers at the same place.

Until about 2000, when actual bed numbers tended to decrease in many HICs, there also was a trend towards lower need estimates. The estimates of appropriate bed numbers decreased over time, possibly because community services had been established that could support patients and provide various treatments outside hospitals. Experts estimating bed needs may have become aware that lower bed numbers have been shown feasible in some countries with mental health reforms and considered these experiences in their estimates. However, the trend to lower need estimates has not been observed from 2000 onwards anymore, at a period when actual bed numbers continued decreasing in different parts of the world, i.e. in Western Europe (Chow & Priebe, 2016), Central and Eastern Europe (Mundt et al., 2021b), Africa (Mundt et al., 2022a), and Latin America (Mundt et al., 2024).

The median need estimate of 47 psychiatric beds needed per 100 000 population after the year 2000 is close to the median of 44 beds reported for HICs today (World Health Organization, 2021). The method to establish a needs estimate may be taken as an indicator of the study quality. Estimates based on epidemiological data can be considered higher in quality than normative reports, and expert consensus higher than expert opinion. However, the epidemiological estimates also have limitations. The models used to calculate bed needs often reduce complex processes and use only simplified indices, such as average waiting times in emergency departments (La et al., 2016). While the estimates based on epidemiological data show more variability and may have higher local validity, other methods arrived at rather consistent estimates across countries or regions. This may indicate some generalizability and provide a guide for regions where there are no reliable epidemiological data on which to base an estimate. Several epidemiological estimates considered bed needs for involuntary treatments. In the public mental health system in Queensland about one-half of inpatient episodes were involuntary (Harris et al., 2012). The state hospital in North Carolina, which also received referrals of patients with complex needs from general hospital psychiatric units, had a majority of involuntary admissions (La et al., 2016). Yet, the discussion on psychiatric bed numbers should be separated from debates about involuntary treatments that need to be embedded in a rights-based approach (Patel et al., 2023).

The scientific literature provides arguments for both further reductions and increases in bed numbers (Mundt, Delhey Langerfeldt, Rozas Serri, Siebenforcher, & Priebe, 2021a). Insufficient integration of community and inpatient care may result in unnecessarily long inpatient stays and call for better continuity of care rather than more beds. Lower costs of services in the community as compared to hospitals have also been mentioned as a reason for further bed reductions. However, in the last 15 years, more publications seem to argue for maintaining or increasing bed numbers than for further reducing them (Mundt et al., 2021a). Different shortcomings in health care services may justify calls for more beds. They include an increase in unplanned admissions, relapse and readmission rates (Ose et al., 2018); higher occupancy levels with overcrowding (Allison et al., 2018; Jeppesen, Christensen, & Vestergaard, 2016); and psychiatric patients gathering in emergency settings (Baia Medeiros, Hahn-Goldberg, Aleman, & O'Connor, 2019; Nordstrom et al., 2019). There are also adverse patient outcomes that may be seen as a consequence of too low bed numbers: an increase in homelessness (Allison et al., 2018); premature mortality; suicides (Bastiampillai, Sharfstein, & Allison, 2016; Hunt et al., 2014); violent crime; and higher patient numbers in prisons (Fuller Torrey et al., 2014; Mundt et al., 2015; Yoon, Domino, Norton, Cuddeback, & Morrissey, 2013). The gap of estimated bed needs and actual bed numbers identified in this review may be seen as consistent with the hypothesis that psychiatric beds are underprovided in most places.

To our knowledge, this is the first systematic review on need estimates of psychiatric beds. It reflects publications from different world regions, albeit dominated by information from HICs. The data cover a large timespan, and more recent data have been synthesized in the analysis. The study also shows how need estimates differ from actual bed numbers.

The study also has several limitations. Our comparison of bed need estimates over different time periods and across countries did not consider the varying contexts such as differences in the quality of out-patient care. The criteria for the different lengths of stay were not always exactly defined in the reviewed literature. Several publications included medium-stay beds, which lacked a clear definition and which were typically collapsed with short or long-stay beds (i.e. short- to medium-stay or medium- to long-stay). The lack of precise definitions of psychiatric beds limits international comparisons and the interpretation of international differences in their provision (Guaiana et al., 2019; O'Reilly et al., 2019; Pinals & Fuller, 2017; Pirkis, Harris, Buckingham, Whiteford, & Townsend-White, 2007). Also, the terminology has changed over time, reflecting ongoing reform processes since the 1950s (Thornicroft, Deb, & Henderson, 2016). Because of the lack of clear and overall accepted definitions similar mental health facilities have been classified differently in different regions or countries (Guaiana et al., 2019). This terminological problem also applies to other mental health interventions, such as inpatient and outpatient treatment in acute, transitional, rehabilitative, and long-term settings (Pinals & Fuller, 2017). The variability of services, the absence of a consistent and overall agreed terminology, and difficulties in establishing which exact populations are cared for in psychiatric inpatient units, all complicate comparisons across countries and time periods (Johnson, Kuhlmann, & EPCAT group, 2000). Whilst these issues also affected our review, they are unlikely to have fundamentally influenced the main results. Finally, there was a scarcity of studies coming from LMICs, which makes it difficult to draw conclusions for those countries.

The need estimates were commonly higher than the actual provision of beds. Historically until 2000, need estimates have moved toward lower numbers of inpatient beds, which may be linked to an increased provision of community-based care or an adaptation of norms and recommendations to actually reduce bed numbers or both. Yet, there is a gap between estimates of what is needed and actually provided. This gap may reflect a worsening under-provision of psychiatric inpatient facilities, which – as inpatient admission is often a last resort in the care of patients posing a risk to themselves or others – may have serious implications and increase adverse outcomes. For some countries, particularly those with very low bed numbers, this may be a reason to revise current policies if they encourage further reductions of bed numbers.

## Supporting information

Mundt et al. supplementary material Mundt et al. supplementary material

## Data Availability

No original data were used in this review.

## Supplementary material

The supplementary material for this article can be found at https://doi.org/10.1017/S0033291724002307.
