# Supplementary material for: Need estimates of psychiatric beds: a systematic review and meta-analysis
Source: Psychol Med. 2024 Sep 26;54(14):3795–808. doi: 10.1017/S0033291724002307 (PMC11578909; doi:10.1017/S0033291724002307)
Supplement: Mundt et al. supplementary material [file S0033291724002307sup001.pdf]

Online supplement, estimates of psychiatric bed needs

Supplement Table 1:

Supplement Table 1. Number of studies per country, categorized by World Bank Income group of the country.

Income group	Country	Studies
**High-income**	United Kingdom	24
	USA	12
	Australia	8
	Canada	6
	Germany	2
	Denmark, Ireland, Japan, Netherlands, New Zealand, Norway, Spain, Switzerland*	1
**Low- and middle-income**	Brazil	2
	South Africa	1
	International	3
**Total**		66

**One study for each country.*

Supplement Figure 1: Need estimates of psychiatric beds with unspecified length of stay per 100 000 population

Year	Study	Bed prevalence with 95% CI
1958	Strömgren	250 [245, 255]
1961	Tooth & Brooke	180 [179, 181]
1964	Gore et al.	193 [186, 200]
1965	Maryland Board of Healh and Mental Hygiene	375 [369, 381]
1965	Ohio Department of Mental Hygiene and Correction	280 [277, 283]
1965	Richman & Kennedy	197 [195, 199]
1966	Valdés	200 [198, 202]
1968	California Department of Mental Hygiene	65 [64, 66]
1969	Oldham	134 [120, 148]
1971	Wing	50 [49, 51]
1972	Lawson	180 [178, 182]
1972	Lipscomb	50 [12, 88]
1972	Mezey & Syed	99 [97, 101]
1973	Muller	100 [100, 100]
1974	Fryers	230 [224, 236]
1976	Straathof	177 [175, 179]
1981	Robertson	140 [139, 141]
1984	Trant et al.	100 [97, 103]
1987	Häfner	110 [110, 110]
1990	Andrews et al.	35 [35, 35]
1996	WHO	10 [10, 10]
1997	Gordon	50 [49, 51]
1998	Dillard et al.	40 [39, 41]
1998	Disley et al.	41 [39, 43]
2006	Lund & Flisher	38 [37, 39]
2007	Boardman & Parsonage	48 [47, 49]
2007	Leite Gastal et al.	50 [50, 50]
2008	Miklebust et al.	100 [97, 103]
2008	Fuller Torrey et al.	50 [50, 50]
2010	Candiago et al.	22 [20, 24]
2011	Tyrer	47 [46, 48]
2012	Harris et al.	30 [28, 32]
2016	La et al.	39 [38, 40]
2018	Allison et al.	50 [50, 50]
2019	Victoria's Mental Health System	51 [49, 53]
2020	Hudson	46 [45, 47]
2021	Hudson	35 [35, 35]
2022	McBain	51 [51, 51]
2022	Mundt et al.	45 [45, 45]
	**Overall**	102 [76, 129]
	Heterogeneity: $I^2 > 99\%$	

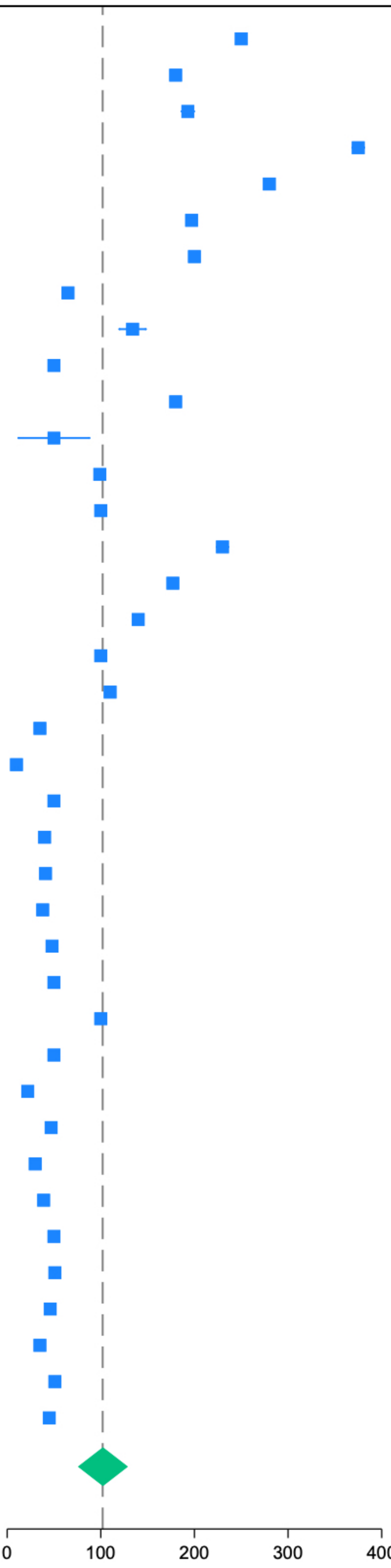

Supplement Figure 2: Need estimates of psychiatric short-stay beds per 100 000 population

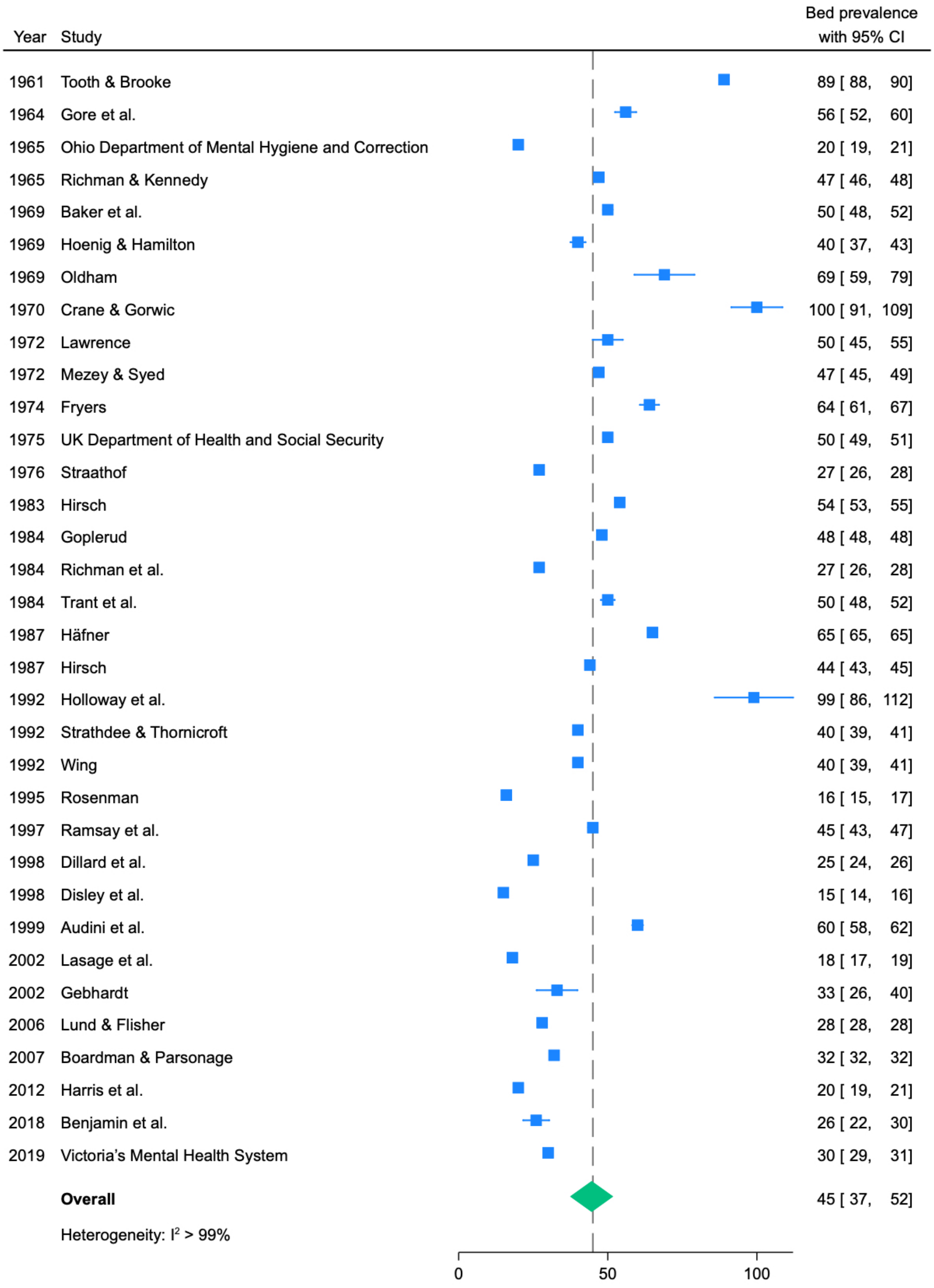

Supplement Figure 3: Need estimates of psychiatric long-stay beds per 100 000 population

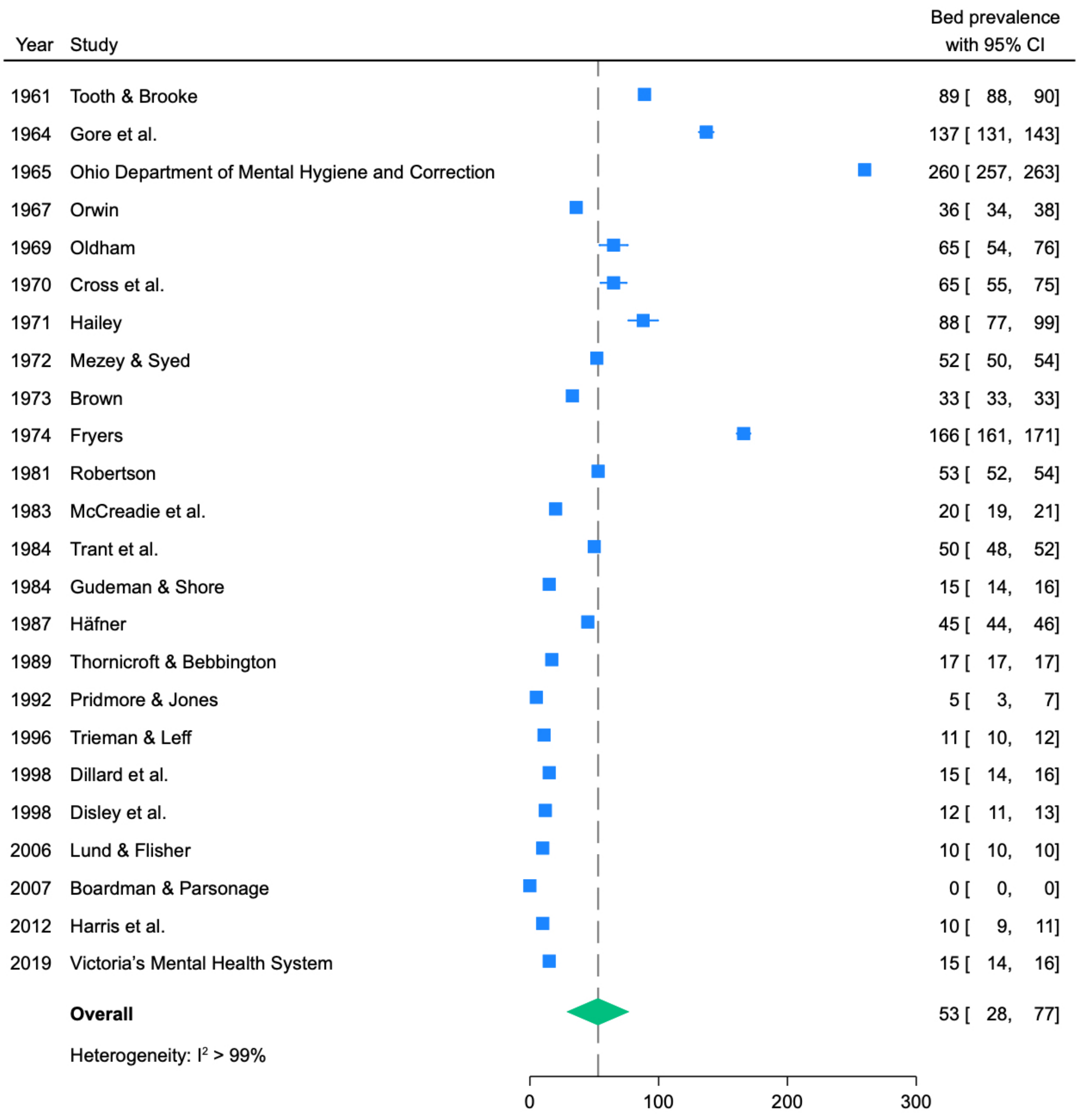

Supplement Figure 4: Trendlines of need estimates for psychiatric beds per 100 000 population over time.

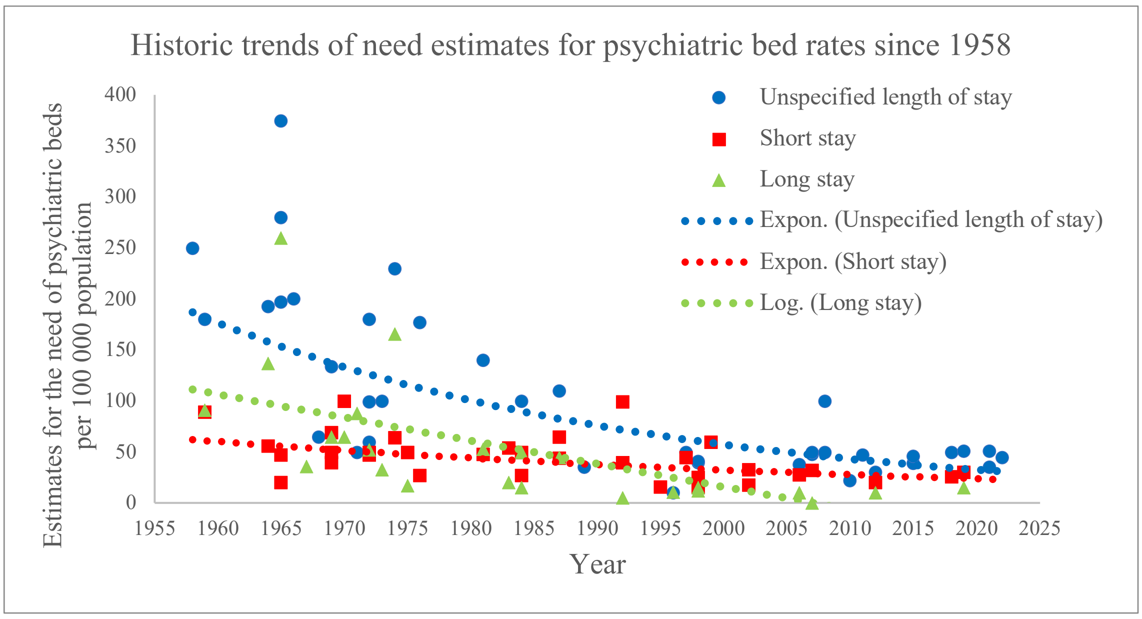

Supplementary Figure 5: Trendlines of need estimates for the psychiatric bed prevalence from 2000 onwards.

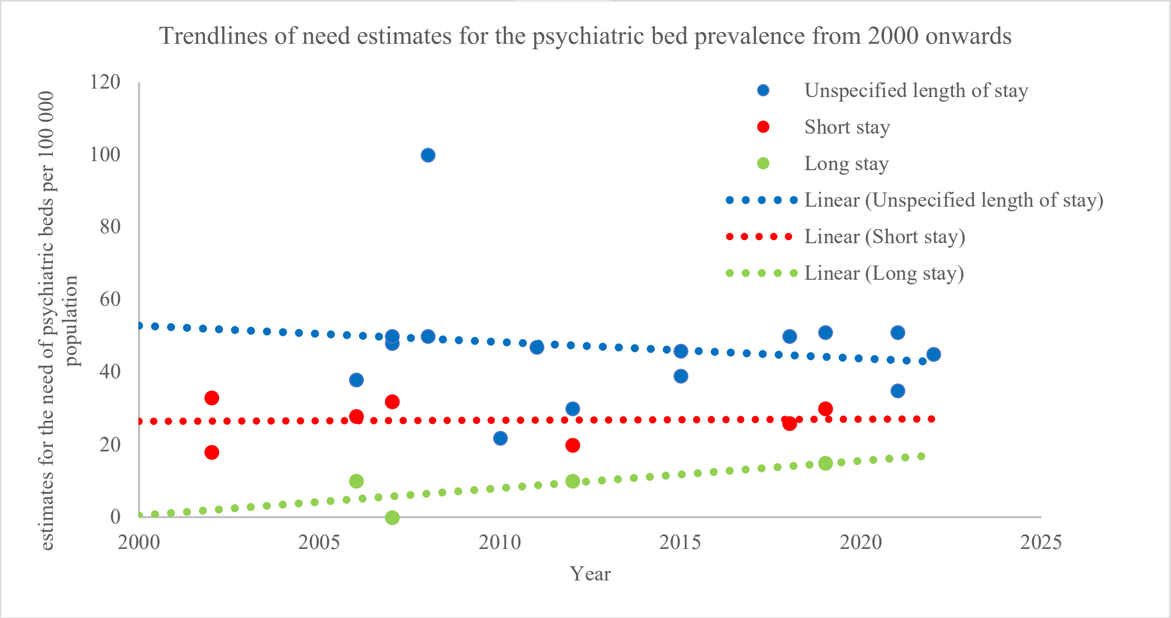

Supplement Figure 6: Prevalence ratios between need estimates and actually available psychiatric beds by length of stay.

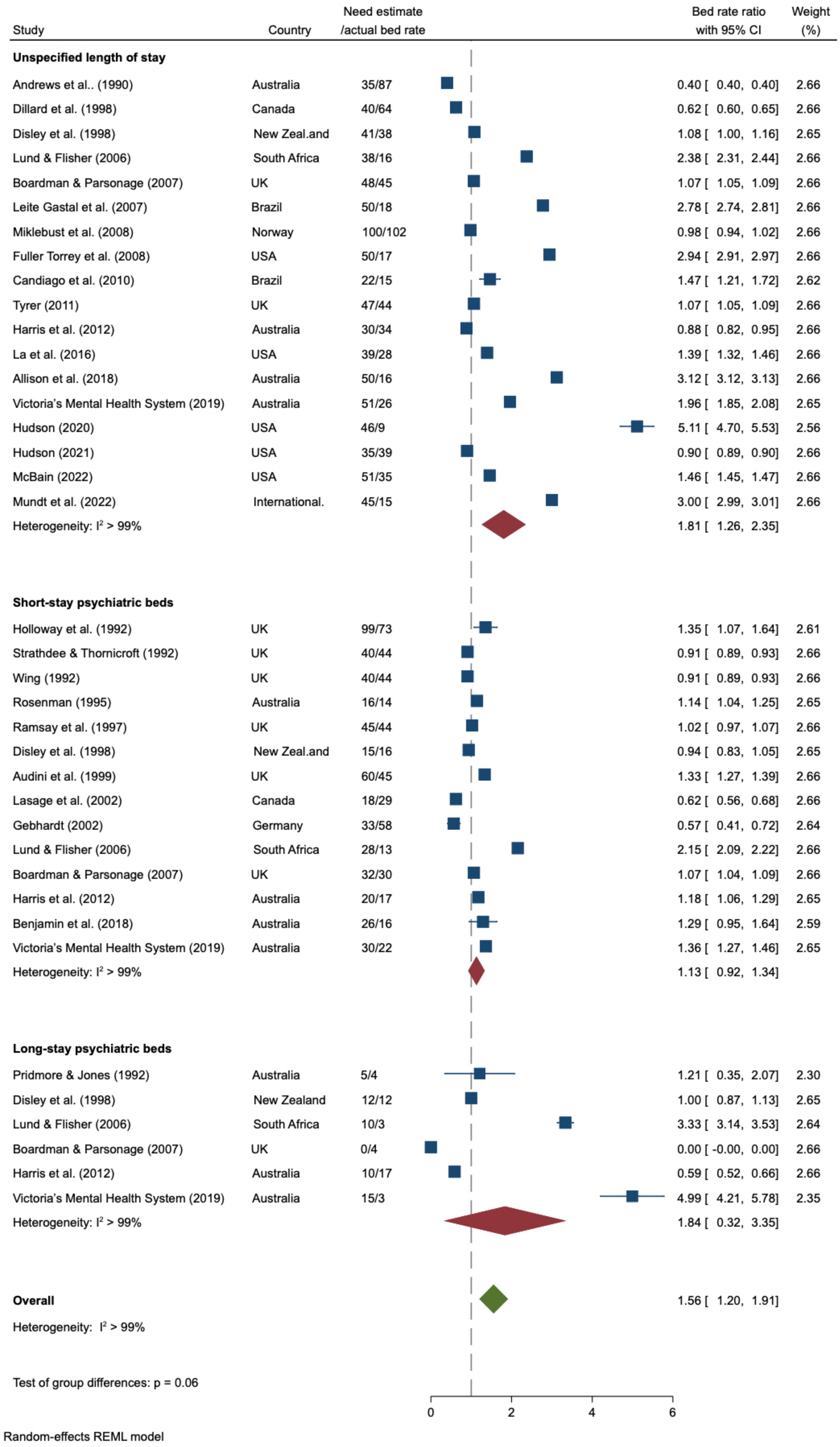

Supplementary Figure 7: Linear trendlines for prevalence ratio between estimates of psychiatric bed needs and actual availability over time (from 1990 onwards) on a semi logarithmic scale. Prevalence ratios smaller than 1 indicate recommendations to remove, and greater than 1, recommendations to increase psychiatric bed numbers. Lines are curved due to the logarithmic scale.

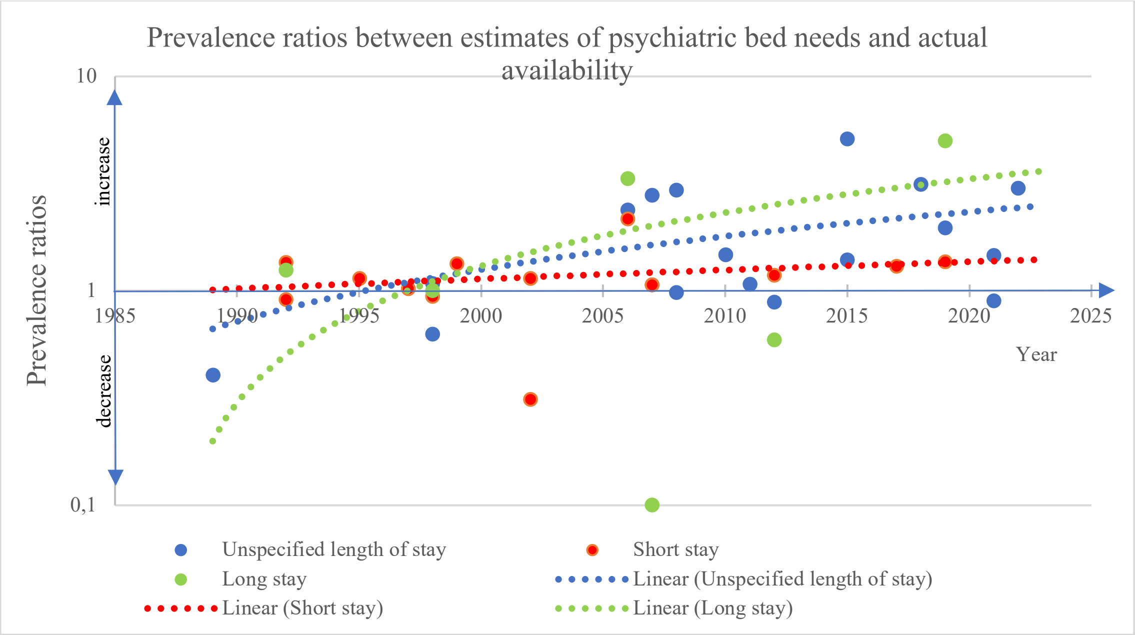

Supplementary Table 2: Quality appraisal of the scientific literature reporting need estimates for psychiatric beds.

	**Supplement Table 2. Quality appraisal of publications.**							
	Author (year of publication)	***Published in indexed journal with peer review***	***Recommendation based on original epidemiological data***	***Primary aim of the study was the recommendation of bed numbers***	***Clear definition of length of stay***	***Provided as prevalences of beds per population***	***Provided as a single number (vs range)***	***Definition of recommendation as "minimal"[a]***
1	*Strömgren E (1958)*	X	X	X		X	X	X
2	*Tooth GC & Brooke E (1961)*	X	X	X	X	X	X	
3	*Gore et al (1964)*	X	X	X	X			
4	*Richman & Kennedy (1965)*	X	X	X	X	X	X	
5	*Ohio Department of Mental Hygiene and Correction (1965)*			X	X	X	X	
6	*Maryland Board of Health and Mental Hygiene (1965)*			X		X	X	
7	*Valdés AJ (1966)*	X		X		X	X	
8	*Orwin A (1967)*	X	X	X	X	X	X	X
9	*California Department of Mental Hygiene (1968)*			X	X	X		
10	*Baker et al (1969)*	X	X	X	X	X	X	X
11	*Oldham AJ (1969)*	X	X	X			X	
12	*Hoenig J & Hamilton MW (1969)*		X	X	X	X	X	X
13	*Crane & Gorwic (1970)*		X	X	X	X	X	
14	*Cross et al (1970)*	X	X	X	X	X	X	
15	*Hailey (1971)*	X	X	X	X	X	X	
16	*Department Health and Social Security (1971)*			X	X	X	X	X
17	*Mezey & Syed (1972)*	X	X	X	X	X	X	
18	*Lipscomb CF (1972)*	X	X	X	X	X		
19	*Lawrence M (1972)*				X	X	X	
20	*Lawson JS (1972)*	X		X		X	X	
21	*Brown BM (1973)*		X	X		X		
22	*Muller C (1973)*	X		X		X	X	
23	*Fryers T. (1974[8]*	X	X	X	X	X	X	
24	*UK Department Health and Social Security (1975)*		X	X	X	X	X	
25	*Straathof LJ (1976)*	X	X	X	X	X	X	
26	*Robertson G (1981)*	X	X	X	X	X	X	
27	*Hirsch SR (1983)*	X	X	X		X		X
28	*McGreadie et al (1983)*	X	X	X	X	X	X	
29	*Goplerud EN (1984)*	X	X	X	X	X		
30	*Richman et al (1984)*	X	X	X	X	X	X	
31	*Gudeman & Shore (1984)*	X	X	X	X	X	X	
32	*Trant et al (1984)*		X	X	X	X	X	X
33	*Häfner H (1987)*	X	X	X	X	X		X
34	*Hirsch SR (1987)*	X	X	X	X	X		X
35	*Andrews et al (1990)*	X	X			X	X	X
36	*Holloway et al (1992)*	X	X		X	X	X	
37	*Strathdee & Thornicroft (1992)*		X	X	X	X		
38	*Wing JK (1992)*		X	X	X	X		
39	*Pridmore & Jones (1992)*	X	X	X	X	X	X	X
40	*Rosenman S (1995)*	X	X	X	X	X	X	
41	*World Health Organisation (1996)*			X		X	X	
42	*Trieman & Leff (1996)*	X	X	X	X	X		
43	*Gordon A (1997)*			X		X	X	
44	*Ramsay et al (1997)*		X	X	X	X		
45	*Dillard et al (1998)*		X	X	X	X	X	
46	*Disley et al (1998)*		X	X	X	X	X	X
47	*Audini et al (1999)*	X	X	X	X	X		X
48	*Gebhardt (2002)*	X	X	X	X		X	X
49	*Lasage et al (2002)*	X	X	X	X	X	X	X
50	*Lund & Flisher (2006)*	X	X	X	X	X	X	
51	*Boardman & Parsonage (2007)*		X	X	X	X	X	
52	*Leite Gastal et al (2007)*	X				X	X	
53	*Fuller Torrey et al (2008)*			X		X		X
54	*Miklebust et al (2008)*	X	X	X		X	X	X
55	*Candiago et al (2010)*	X	X					X
56	*Tyrer P (2011)*	½[b]		X			X	X
57	*Harris et al (2012)*	X	X	X	X	X	X	
58	*La et al (2016)*	X	X	X			X	
59	*Allison et al (2018)*	X		X		X	X	X
60	*Benjamin et al (2018)*	½[b]		X	X		X	X
61	*Victoria's Mental Health System (2019)*		X	X	X	X	X	
62	*Hudson (2020)*	X	X	X		X	X	X
63	*Hudson (2021)*	X	X	X		X	X	X
64	*McBain et al (2021)*		X	X	X	X	X	X
65	*Mundt et al (2022)*	X		X	X	X		X

[a] "Minimal" considers all reports that refer to the recommendation as "minimal", "sufficient", "at least", "necessary", "bedrock" or "required".
[b] Indexed journal, but format that does not typically undergo peer review, like correspondence
